# Urethrocutaneous fistula following VMMC: a case series from March 2013 to October 2019 in ZAZIC’s voluntary medical male circumcision program in Zimbabwe

**DOI:** 10.1186/s12894-022-00973-1

**Published:** 2022-02-16

**Authors:** Vernon Murenje, Victor Omollo, Paidemoyo Gonouya, Joseph Hove, Tinashe Munyaradzi, Phiona Marongwe, Mufuta Tshimanga, Vuyelwa Chitimbire, Sinokuthemba Xaba, John Mandisarisa, Shirish Balachandra, Batsirai Makunike-Chikwinya, Marrianne Holec, Tonderayi Mangwiro, Scott Barnhart, Caryl Feldacker

**Affiliations:** 1 Zimbabwe Technical Assistance, Training and Education Center for Health (Zim-TTECH), Harare, Zimbabwe; 2 Department of Global Health, University of Washington, Seattle, WA USA; 3 Zimbabwe Association of Church-Related Hospitals (ZACH), Harare, Zimbabwe; 4 Zimbabwe Community Health Intervention Project (ZiCHIRe), Harare, Zimbabwe; 5 Ministry of Health and Child Care, Harare, Zimbabwe; 6 The Centers for Disease Control and Prevention (CDC), Harare, Zimbabwe; 7 International Training and Education Center for Health (I-TECH), Seattle, WA USA; 8 Department of Surgery, University of Zimbabwe College of Health Sciences, Harare, Zimbabwe; 9 Department of Medicine, University of Washington, Seattle, WA USA

## Abstract

**Background:**

Urethrocutaneous fistula (subsequently, *fistula*) is a rare adverse event (AE) in voluntary medical male circumcision (VMMC) programs. Global fistula rates of 0.19 and 0.28 per 100,000 VMMCs were reported. Management of fistula can be complex and requires expert skills. We describe seven cases of fistula in our large-scale VMMC program in Zimbabwe. We present fistula rates; provide an overview of initial management, surgical interventions, and patient outcomes; discuss causes; and suggest future prevention efforts.

**Results:**

Case details are presented on fistulas identified between March 2013 and October 2019. Among the seven fistula clients, ages ranged from 10 to 22 years; 6 cases were among boys under 15 years of age. All clients received surgical VMMC by trained providers in an outreach setting. Clients presented with fistulae 2–42 days after VMMC. Secondary infection was identified in 6 of 7 cases. Six cases were managed through surgical repair. The number of repair attempts ranged from 1 to 10. One case healed spontaneously with conservative management. Fistula rates are presented as cases/100,000 VMMCs.

**Conclusion:**

Fistula is an uncommon but severe AE that requires clinical expertise for successful management and repair. High-quality AE surveillance should identify fistula promptly and include consultation with experienced urologists. Strengthening provider surgical skills and establishment of standard protocols for fistula management would aid future prevention efforts in VMMC programs.

## Background

Voluntary medical male circumcision (VMMC), an elective procedure which provides partial protection from HIV acquisition [1], poses a risk of adverse events (AEs) like other common surgical procedures [1, 2]. Guidance from UNAIDS spurred rapid scale-up of VMMC in high HIV prevalence countries in Eastern and Southern Africa, reaching 4 million VMMCs in 2018, alone [3]. Reported rates of moderate and severe AEs within VMMC programs in sub-Saharan Africa range from 0.1 to 8% [4–9]. Common AEs reported in VMMC programs include infections, bleeding, swelling and pain [4, 10–12], and occasionally severe AEs such as urethrocutaneous fistula (subsequently*, fistula*) and glans amputation [13, 14]. Fistulae are rare in VMMC programs. The World Health Organization (WHO) found 32 cases of fistulae [15] among 16,790,262 reported VMMCs between 2014 and 2018 [16], giving a global fistula rate of 0.19 per 100,000 VMMCs, and a review of U.S. President’s Emergency Plan for AIDS Relief (PEPFAR) VMMC data from 15 countries reported a fistula rate of 0.28 per 100,000 VMMCs between 2015 and 2019 [17].

Fistula is an abnormal passageway between the urethra and the surface of the penile skin that causes urine to pass either partially or entirely through the skin opening, rather than through the normal urethral opening at the tip of the penis. Fistula can result from a cut into the urethra, a deep stitch which pierces or includes part of the urethra, diathermy burns and wound infection [15, 18]. Fistulae commonly occur at the six o’clock position on the distal ventral aspect of the penis where the urethra is closest to the skin. Like other AEs, when fistulae occur, they can lead to costly and time-consuming clinical management; reduced quality of life, possible disfigurement, stigma, and emotional distress for the client; and negative community perception of VMMC [13, 19].

ZAZIC, a consortium of three partners (the International Training and Education Center for Health (I-TECH), Zimbabwe Association of Church related Hospitals (ZACH) and Zimbabwe Community Health Intervention Research Project (ZiCHIRe)), has been implementing VMMC in coordination with the Ministry of Health and Child Care (MoHCC) in Zimbabwe since 2013. The name, *ZAZIC*, is a combination of consortium partner names. Over its 7-year program, ZAZIC circumcised 461,942 men in 55 sites from March 2013 through October 2019. With support from the Centers for Disease Control and Prevention (CDC) and PEPFAR, ZAZIC provided VMMC services to men aged 10 years and above through the forceps guided (FG) and dorsal slit (DS) surgical methods as well as non-surgical VMMC via the PrePex device. Use of the FG method in males under 15 years was halted in 2015 in accordance with WHO and PEPFAR guidance, as the method was associated with higher rates of glans injuries [20]. PrePex was discontinued in December 2016 due to tetanus risk [21].

In 2017, ZAZIC requested assistance in the management of ongoing fistula cases from MOHCC and CDC. In response, the WHO Regional Office and WHO Technical Advisory Group on Innovations in Male Circumcision (TAG) held a *Consultation on Management of Urethral Fistula Adverse Events Following Medical Male Circumcision for HIV Prevention* workshop in Harare, Zimbabwe in October 2019. The workshop covered fistula risk, prevention and management discussions followed by practical sessions of fistula repair and skills transfer led by two international, expert urologists from India. Public health specialists, program managers and clinical specialists from 11 African countries, the UK, India, USA, Switzerland and India participated.

Since fistulae are rare and there is limited literature of cases in VMMC programs, we present our fistula rate and descriptive case series on seven fistulae that occurred in the ZAZIC-supported VMMC sites from 2013 to 2019. The objective of the case series report is to describe cases of fistulae, identify potential root and contributing causes, describe case management and guide potential interventions to prevent future fistulae.

## ZAZIC AE surveillance system

**Fig. 1:**
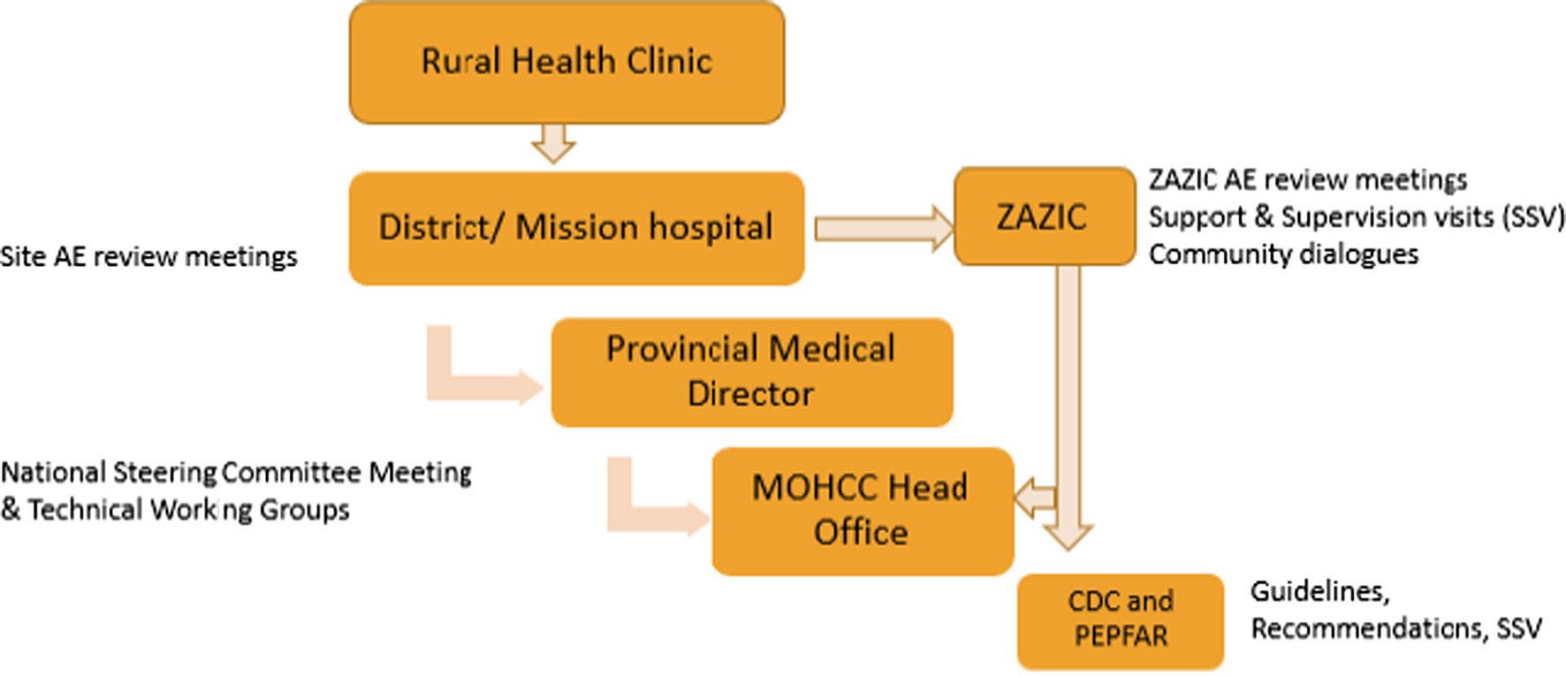
AE reporting [22]

Severe AEs, like fistula, are reported to MoHCC and to CDC Zimbabwe within 48 h as per PEPFAR guidelines following a path shown in Fig. 1. AEs from outreach and clinics are reported to district hospitals. There, one copy of the severe AE report is sent to the Provincial Medical Directorate and to MoHCC head office and another to the implementing partner, ZAZIC. ZAZIC reports to CDC. Reviews of reported AEs and subsequent actions are deliberated at site, partner and at national level through the National VMMC Steering Committee and Service Delivery and Training Technical Working Groups. Demographic details, management procedures and outcomes for the fistula case reports were collected from a retrospective review of submitted ZAZIC program AE reporting forms and updates.

## Case reports

Table 1 summarizes the cases of urethrocutaneous fistula encountered within the ZAZIC VMMC program.

**Table 1:** Summary of urethrocutaneous fistulae cases reported by ZAZIC

Case	1	2	3	4	5	6	7
Year of VMMC	2013	2014	2015	2016	2017	2019	2019
Age (years)	22	14	10	11	12	12	13
Circumcision method	Forceps guided	Forceps guided	Forceps guided	Dorsal slit	Dorsal slit	Dorsal slit	Dorsal slit
Site	Outreach						
Circumcising cadre	Doctor	Doctor	Doctor	Nurse	Nurse	Nurse	Nurse
Surgeon experience in VMMC	1 year	2 years	2 years	4 years	4 months	6 years	5 years
Suture material/needle size	3/0 vicryl, 26 mm, 3/8 circular reverse cutting needle						
Diathermy used	No						
Days to fistula diagnosis after VMMC	2	7	15	42	18	17	14
Number of repair attempts	10	3	None	3	3	2	1
Surgeon expertise and repair dates (month/year)	*GP #1-Aug 2013 GP #1-Aug 2013 GP #1-Aug 2013 GP #1-Sept 2013 Urologist #1-May 2014 Urologist #1-Nov 2014 Urologist #1-May 2015 Urologist #2-November 2015 Urologist #1 and #2-Mar 2017 WHO Urologists-Oct 2019	General Surgeon #1-May 2015 Urologist #2-Oct 2016 Urologist #2-Jan 2018	Conservative management by GPs #2 and #3- March 2015	General Surgeon #1-Jan 2017 Urologist #2-Jan 2018 Urologist #1-Sept 2020	Urologist #2-Jun 2017 Urologist #2-Nov 2017 W.H.O Urologists- Oct 2019	Urologist #1-May 2019 Urologist #1-Sept 2020	Urologist #1-Mar 2020
Days to healing	2241	1830	21	1413	940	244	196
Secondary diagnosis	Infection	Infection	Infection	None	Infection	Infection	Infection
Outcome	Healed	Healed	Healed	Healed	Healed	Unhealed	Healed

^*^A general practitioner (GP) in Zimbabwe is a licensed primary care physician who refers clients appropriately for specialized care. Provider # is a unique ID, e.g., Urologist #1 is the same provider across clients

### Case 1

In 2013, a 22 year old male was circumcised at an outreach site by a trained physician with 1-year experience through the FG method and no diathermy. At the day 2 post-VMMC review, a gap was noted on the wound with urine leaking. The client was referred to a district hospital where he was reviewed by a Government Medical Officer (general practitioner (GP)) who diagnosed fistula on the ventral side of the penis around the frenulum; the client was discharged home on daily saline baths and scheduled for repair after 2 weeks. Four unsuccessful repair surgeries were subsequently performed by the GP within 6 weeks of hospital stay; in each of the 4 procedures, the GP placed sutures too close the fistula. Nine months after the circumcision, a consultant urologist reviewed the client and noted extensive scarring and fibrosis. Five additional repair attempts were made by two different urologists over the next 47 months. Each repair appeared initially successful but ultimately resulted in postoperative infection where the sutures broke down and the fistula started leaking again. The tenth repair was performed by WHO specialists in 2019 (2244 days post VMMC). The repair was successful. Client healed completely.

### Case 2

In 2014, a 14 year old male was circumcised at an outreach site by a trained physician with 2 years’ experience using the FG method and no diathermy. 2-day post-VMMC review noted the client had poor hygiene and he received post-operative wound care counseling. The client presented on day seven post-VMMC with complaints of pus oozing from the frenulum. Wound cleaning was done using iodine solution and the client was given oral Amoxicillin 500 mg for seven days. On day ten post-VMMC, the client noted little improvement and returned to the clinic. Examination revealed pus oozing from the frenulum with urine leaking on the ventral aspect of the penis. A diagnosis of fistula was made. The client was admitted to hospital where he received Ceftriaxone 1000 mg intravenously for 1 week and Metronidazole 500 mg for 2 weeks and a urethral catheter was inserted. The client was later reviewed and discharged by a general surgeon who referred him to an urologist. Reconstructive surgery was postponed several times due to other emergency cases. After several further postponements of the surgery, the client’s parents lost confidence and stopped attending further reviews. In early 2015, the parents were contacted and offered an option of a private practitioner for the repair, but they refused until late 2015 (525 days post-VMMC) when they consented to surgery and the first repair was done by a general surgeon. He was discharged after 2 weeks post repair operation with a urethral catheter which was removed after 6 weeks. On removal of the catheter a pinhole size fistula was noted and the surgeon was hopeful the wound would close spontaneously. The client was again reviewed in early 2016 and urine was still leaking. He was booked for another surgery but the parents did not consent. Later in 2016 the client presented with history of passing blood in urine and was referred to an urologist. A diagnosis of bladder stone was made and the urologist removed the stone and performed a second fistula repair during the same operation (914 days post-VMMC), closing the wound in two layers before placing both suprapubic and urethral catheters for 10 days. In early 2017, the client reported that the fistula had started leaking again. A team of two urologists reviewed him and observed a pinhole fistula but deferred repair to allow for spontaneous closure. He was reviewed 6 months later and the fistula was noted to be still leaking. The third fistula repair in early 2018 (1355 days post-VMMC) resulted in a wound infection 1-month post repair operation; the sutures broke down and the fistula reopened. After conservative management and observation, a review done after about 16 months (1832 days post-VMMC) found that the fistula had closed spontaneously.

### Case 3

In 2015, a 10 year old male was circumcised at an outreach site by a trained physician with 2 years’ experience using the FG method and no diathermy. Day 2 and day 7 reviews were reportedly uneventful. The client presented on day 15 post-VMMC with a complaint of urine leaking around the wound. On examination, the wound looked infected and three fistulae openings were noted with urine leaking on the ventral aspect of the penis around the frenulum. Immediate management by two Government Medical Officers (GPs) involved cleaning with iodine solution, catheterization for 2 weeks, and intravenous Ceftriaxone for seven days. The client was seen by a general surgeon 2 weeks after the initial management who noted that there was no urine leakage upon removal of the catheter. The general surgeon confirmed fistula healing at 6 the weeks post-VMMC review.

### Case 4

In 2016, an 11 years old male was circumcised at an outreach site by a trained nurse with 4 years’ experience, using the DS and no diathermy. Normal findings were reportedly observed on day 2 and day 7 reviews. The client presented at the clinic on day 42 post-VMMC complaining of passing urine from the ventral and lateral aspect of the penis. On examination, a small opening was noted on the ventral aspect of the penis without leakage while another opening on the right lateral side of the penis along the circumcision suture line was observed to be leaking urine. A diagnosis of fistulae was made. On day 73 post-VMMC, a General Surgeon confirmed urine leakage from the two openings and planned for repair which was done on day 110. Two months after the repair, the client was reviewed by a team of two urologists who observed that the fistula was pinpoint in size, not leaking and gave the fistula a chance to close spontaneously. Approximately 1 year after the first repair, the client presented again at the clinic with complaints of urine leakage, and a second repair attempt was completed (day 473 post VMMC). The second repair attempt was not successful, and client continued to leak urine. Follow-up surgery was deferred for the WHO workshop. However, the client’s school obligations prevented participation. A third repair by a local urologist who completed the WHO workshop was performed in September 2020. A review at 2 weeks post repair operation (day 1462 post-VMMC) and review 2 months post repair operation confirmed healing.

### Case 5

In 2017, a 12 year old male was circumcised at an outreach site by a trained nurse with 4 months’ experience, using the DS method and no diathermy. Normal findings were reportedly observed on day 2 and day 7 reviews. On day 18 post-VMMC, the client noticed urine leaking on the underside of the penis. He presented to the clinic two days later where a clean wound with some urine drops leaking on the frenulum along the suture line was observed. A diagnosis of fistula was made, and the client was referred to an urologist at tertiary hospital a day later. He was admitted, catheterized and started on Ceftriaxone 1 g intravenously for a week. The client was discharged to an outpatient clinic where he continued with daily wound cleaning using iodine solution. At day 61 post-VMMC, the wound was clean but the fistula was still leaking. The client continued on a urethral catheter until 92 days post-VMMC, when a surgical repair was attempted. A 1-month post-repair review (day 122 post-VMMC) found a small opening that was leaking urine. A second surgical repair at 270 days post-VMMC resulted in post-operative infection: sutures broke down and the fistula started leaking. A third and successful fistula repair was done by WHO specialists 2 years after the second repair (961 days post-VMMC).

### Case 6

In 2019, a 12 year old male was circumcised at an outreach site by a trained nurse using the DS method and no diathermy. Normal findings were reportedly observed on day 2 and day 7 reviews. On day 17 post-VMMC, the client presented to the clinic complaining of urine dripping from the underside of the penis. He reported that he had stopped saline baths for wound cleaning and was, instead, applying Cotrimoxazole tablets provided by his grandmother directly on the wound. On examination, a septic wound was noted on the frenulum and urine was leaking through the wound. A diagnosis of fistula was made and the client was admitted to hospital under the care of a GP for wound cleaning, insertion of urethral catheter, and Amoxicillin Clavulanate 375 mg for a week. He was referred to an urologist who advised to keep the catheter for a further 3 weeks. After the three weeks, the catheter was removed and the fistula was still open. An attempt at repair was done by a team of two urologists 135 days post-VMMC. Upon removal of the catheter 1 month later, the urologists noted that the fistula had closed. Six months post first repair, the client again reported urine leaking. A second repair was performed by an urologist in September 2020. Review 2 weeks later by an urologist (244 days post-VMMC) suggested healing. Six weeks later, a VMMC nurse observed urine leakage during a scheduled review and referred client back to an urologist. Client was reviewed by the urologist 2 months after repair operation who confirmed a recurrent pinhole fistula at 3 o’clock position. Surgical repair of the recurrent fistula was postponed to allow spontaneous healing. The fistula opening remains pinhole-sized as per clinical examination in September 2021.

### Case 7

In 2019, a 13 year old male was circumcised at an outreach site by a trained nurse with 5 years’ experience using the DS method and no diathermy. The client did not attend day 2 and day 7 reviews. Client follow up was not conducted due to an incomplete client address captured at enrollment. He presented to the clinic on day 14 post-VMMC, complaining of pain and urine leaking from near his circumcision wound. Examination showed wound dehiscence and a fistula on the ventrolateral (4 o’clock) position. Immediate management by a Government Medical Officer included wound cleaning, oral Amoxicillin Clavulanate 625 mg and Paracetamol 1000 mg for a week. The client was reviewed on day 28 post-VMMC by an urologist who recommended continued wound care. Reviews at both day 41 and day 55 post-VMMC, revealed a clean wound which was healing well but with urine still leaking. On day 161 post-VMMC, the urologist performed a fistula repair and a urethral catheter was left in situ. Amoxicillin Clavulanate 625 mg was administered for a week and Mupirocin ointment applied for 2 weeks with alternate days of cleaning and dressing for the initial week. At 23 days post repair (day 184 post VMMC), the urologist review concluded that the fistula had completely healed.

### Fistula rates

Over 6 years, fistula cases were reported at 5 of 55 (9%) ZAZIC facilities (Table 2). One facility reported 3 fistula cases resulting from 3 different providers over a 5-year period. Site fistula rates ranged from 4.2 to 12.2 per 100,000 VMMCs, see Table 2.

**Table 2:** ZAZIC site fistula rates

	Site A	Site B	Site C	Site D	Site E	Global (WHO data)	Global (PEPFAR data)
# of Fistulae	1	1	1	3	1	32	41
*Total VMMCs/site	15 737	23 711	8 005	34 198	8176	**16 790 262	***14 900 000
Fistula rate per 100,000 VMMCs	6.4	4.2	12.5	8.8	12.2	0.19	0.28

^*^ZAZIC VMMCs, March 2013–October 2019

^**^WHO Total Global VMMCs, 2014–2018

^***^PEPFAR Total Global VMMCs, 2015–2019

## Discussion

Within the ZAZIC program, a total of 7 cases of fistulae were reported between 2013 and 2019, reflecting a fistula rate of 1.52 per 100,000 VMMCs. This is higher than the reported rates from either WHO or PEPFAR that reported 0.19 and 0.28 fistulas per 100,000 VMMCs, respectively. Although fistulae are rare in VMMC programs, ZAZIC’s reported fistula rate was 5–eightfold higher than global reports. We suggest that ZAZIC’s higher fistula rate potentially reflects attention to AE ascertainment, strengthened reporting, and complete documentation processes rather than from safety issues specific to the ZAZIC program. However, given that treatment for several clients was prolonged and required multiple surgical interventions, ZAZIC is strengthening its routine reviews of program AEs to identify root causes and implement site- or provider-specific training and mentorship to improve the quality of MC procedures. We note the potential causes of fistulae in the ZAZIC program and actions that will reduce future fistula cases.

Our experience managing fistulae demonstrates that early identification, swift escalation to specialist urologists for consultation, and collaboration with an international technical team of experts was most effective in fistula management. Participation in the WHO Zimbabwe fistula workshop led to several changes in ZAZIC and MoHCC fistula care processes. First, fistulae can be managed through surgery or conservative management (waiting for spontaneous resolution) [23, 24]. Initially, conservative management may be advised instead of early repair to allow adequate tissue granulation and complete resolution of any infection, as the first repair is the most likely to succeed [17, 25, 26]. MoHCC recommended in 2016 that all cases of fistulae be referred to general surgeons or specialist urologists and the WHO workshop experts suggested engagement of specialist urologists with experience in urethroplasty for initial repair. A mandatory waiting period before repair following any new diagnosis of fistula or failed surgical repair would allow the tissue around the wound to clearly demarcate and allow scars to mature and help reduce induration and oedema [25]. Lastly, during VMMC procedures, a WHO consultative meeting [27] suggested assurances of adequate lighting in operating rooms, adopting clinician quotas to avoid fatigue, and referral of younger boys to more experienced providers.

Patient factors may also contribute to fistula development. Six of 7 fistula cases were among 10–14 year old clients. Adolescent boys are at a higher risk of serious AEs including rare AEs like fistula and glans injuries due to immature genitalia which predispose young boys to risk of injury during circumcision procedures [4, 13, 17, 26]. Recent guidelines to limit circumcisions to males 15 years and older for safety and consent concerns [26, 28] may be the most effective control measure to reduce risk of fistula in VMMC programs. Secondly, infection was diagnosed concomitantly with fistula in six of seven cases. Infection may follow tissue injury during surgery [15] and younger boys are more likely to have infections due to poor wound care [29, 30]. ZAZIC has strengthened VMMC postoperative wound care in several ways. ZAZIC provides wound care instructions to younger boys in the presence of guardians with simplified, pictorial wound care instructions. VMMC postoperative wound care instructions are also discussed and taught at community gatherings during VMMC mobilization to enhance community knowledge.

Although VMMC AE rates decrease with increasing level of provider experience [31, 32], provider experience in VMMC did not appear to be a risk factor for urethrocutaneous fistula. With the exception of one case which was conducted by a clinician with only 4 months of experience, all circumcisions were conducted by clinicians with more than 1 year of experience. Similarly, provider type did not appear to contribute to the AEs. The seven cases were almost distributed equally between circumcising doctors and nurses, echoing previous studies that found no difference in safety between doctor and nurse VMMC practice [8, 33]. However, 3 cases came from the same district hospital, suggesting that training and supervision may play a role. To ensure adequate supervision and quality care, ZAZIC Quality Assurance officers were deployed following each reported fistula to conduct root cause analysis and refresher training. Practical sessions on VMMC DS surgical skills included review of surgical techniques to prevent urethrocutaneous fistula, practice of mandatory V-neck cut around frenulum, identification and ligature of individual bleeders to avoid multiple sutures around the frenulum area and lifting of skin edges during suturing to avoid going too deep with the needle. ZAZIC also strengthened VMMC postoperative follow up through provision of additional field vehicles to trace clients and expanded tandem reviews [34] to ensure high quality VMMC postoperative reviews, including accurate identification, correct management, and swift reporting of AEs. ZAZIC continues to reassure service providers that AEs will occur and that AE reporting is a sign of quality care provision. To incentivize accurate reporting of all AEs, in 2019, ZAZIC awarded a prize to the site with the most number of AEs documented accurately in recognition of quality reporting.

Program factors, including the type and quality of suture material used, may contribute to fistula development. If the needle is blunt and excessive force is applied, providers may accidentally suture too deep resulting in urethral tissue injury. Injury of the urethra, which can result in fistula, is more likely to occur when providers place many sutures to control bleeding around the frenulum where the urethra is close to the skin [15]. To mitigate risk, WHO issued a recommendation in December 2019 for VMMC programs to decrease needle size and change suture material used in VMMC from 3.0 vicryl suture on a 26 mm 3/8 circle reverse cutting needle to a 4.0 vicryl suture on a 19 mm, 3/8 circle, reverse-cutting needle. Also, all fistulae occurred in males who were circumcised in outreach facilities (small clinics, schools, or public spaces) where VMMC teams from static sites usually pitch tents or caravans to provide VMMC services closer to rural populations. Static facilities typically have better equipment, spacious operating rooms with better lighting and more trained staff whereas equipment shortages and potential non-sterile environments contribute to higher AE rates in outreach settings [14]. With about 75% of all ZAZIC VMMCs occurring at outreach facilities, worksite constraints and fatigue from travel may contribute to urethrocutaneous cases. Standards for service delivery hours that cap outreach event times, ensure adequate equipment, and rotate clinic teams could improve outreach VMMC quality and reduce staff fatigue.

### Limitations

Provider experience was expressed as years of VMMC professional experience, rather than the actual number of clients circumcised. This may not accurately reflect provider experience in cases where providers may be inactive for long periods. Data was not available on staffing levels, number of clients circumcised on day of event VMMC, time of circumcision, all which may point to fatigue towards the end of the day. The analysis did not focus on detailed clinical aspects of the cases including fistula size, surgical techniques during repair, or postoperative management which may inform future surgical management of urethrocutaneous fistulae. Lastly, although there is always a risk of AE underreporting [34], severe AE reporting within the ZAZIC program is quite good, reducing risk of underreporting of fistula cases.

## Conclusion

As VMMC programs continue to push to meet targets, program safety should remain a priority. New guidance, limiting VMMC to boys 15 years and older, should reduce fistula cases. Increased emphasis on AE surveillance, emphasizing complete and accurate reporting, is needed to detect rare and severe AEs such as fistula. When fistula is identified, expert urologists should perform repairs and manage healing, taking care to allow for initial healing and not rush to repair. Strengthening surgical skills through continuous refresher trainings for all providers, enhanced VMMC postoperative wound care for younger males, and establishment of standard protocol of management of fistulae by VMMC programs should be implemented. Consideration should also be given to ensuring adequate site resources to minimize the potential for an AE, including provider rest time to reduce fatigue and proper suture materials.

## Data Availability

The datasets generated during and analysed during the current study are not publicly available due to Ministry of Health and Child Care data restrictions and confidentiality protections. Data may be made available from the corresponding author on reasonable request.

## Abbreviations

VMMC: Voluntary Medical Male Circumcision
Zim-TTECH: Zimbabwe Technical Training and Education Center for Health
ZACH: Zimbabwe Association of Church-related Hospitals
ZiCHIRe: Zimbabwe Community Health Intervention Project
MoHCC: Ministry of Health and Child Care, Harare Zimbabwe
CDC: The Centers for Disease Control and Prevention
I-TECH: International Training and Education Center for Health
PEPFAR: President's Emergency Plan for AIDS relief
AE: Adverse Event
UNAIDS: The Joint United Nations Programme on HIV/AIDS
WHO: The World Health Organization
FG: Forceps Guided
DS: Dorsal Slit
TAG: WHO Technical Advisory Group on Innovations in Male Circumcision
UK: The United Kingdom
USA: The United States of America
SSV: Support and Supervision Visit
GP: General practitioner
